# Sex differences and sex steroids influence on the presentation and severity of cardiovascular autonomic neuropathy of patients with type 1 diabetes

**DOI:** 10.1186/s12933-023-01766-y

**Published:** 2023-02-15

**Authors:** Lía Nattero-Chávez, María Insenser, Alejandra Quintero Tobar, Elena Fernández-Durán, Beatriz Dorado Avendaño, Tom Fiers, Jean-Marc Kaufman, Manuel Luque-Ramírez, Héctor F. Escobar-Morreale

**Affiliations:** 1 Department of Endocrinology and Nutrition, Hospital Universitario Ramón y Cajal, Carretera de Colmenar, Km 9.1, 28034 Madrid, Spain; 2 Diabetes, Obesity and Human Reproduction Research Group, Instituto Ramón y Cajal de Investigación Sanitaria (IRYCIS), Centro de Investigación Biomédica en Red de Diabetes y Enfermedades Metabólicas Asociadas (CIBERDEM), Instituto de Salud Carlos III, Madrid, Spain; 3 Laboratory for Hormonology and Department of Endocrinology, Ghent University Hospital, 9000 Ghent, Belgium; 4 University of Alcalá, Madrid, Spain

**Keywords:** Autonomic nervous system, Cardiovascular disease, Cardioautonomic neuropathy, Sexual dimorphism, Sex differences, Sex hormones, Sex steroids, Type 1 diabetes mellitus

## Abstract

**Background:**

Sex differences characterize cardiovascular outcomes in patients with type 1 diabetes. Cardioautonomic neuropathy is a common complication of type 1 diabetes that associates increased morbi-mortality. Data regarding the interplay between sex and cardiovascular autonomic neuropathy are scarce and controversial in these patients. We aimed to address sex-related differences in the prevalence of seemingly asymptomatic cardioautonomic neuropathy in type 1 diabetes, and their associations with sex steroids.

**Methods:**

We conducted a cross-sectional study including 322 consecutively recruited patients with type 1 diabetes. Cardioautonomic neuropathy was diagnosed using Ewing's score and power spectral heart rate data. We assessed sex hormones by liquid chromatography/tandem mass spectrometry.

**Results:**

When considering all subjects as a whole, asymptomatic cardioautonomic neuropathy prevalence was not significantly different between women and men. When age was taken into account, the prevalence of cardioautonomic neuropathy was similar among young men and those > 50 years. However, in women > 50 years, the prevalence of cardioautonomic neuropathy doubled that of young women [45.8% (32.6; 59.7) *vs.* 20.4% (13.7; 29.2), respectively]. The OR of having cardioautonomic neuropathy was 3.3 higher in women > 50 years than in their younger counterparts. Furthermore, women presented more severe cardioautonomic neuropathy than men. These differences were even more marked when women were classified according their menopausal status instead of age. Peri- and menopausal women had an OR 3.5 (1.7; 7.2) of having CAN compared with their reproductive-aged counterparts [CAN prevalence: 51% (37; 65) *vs.* 23% (16; 32), respectively]. A binary logistic regression model (R^2^: 0.161; *P* = 0.001) displayed age > 50 years as a significant determinant of cardioautonomic neuropathy only in women. Androgens were positively associated with heart rate variability in men, and negatively in women. Accordingly, cardioautonomic neuropathy was associated with increased testosterone/estradiol ratio in women but to decreased testosterone concentrations in men.

**Conclusions:**

Menopause in women with type 1 diabetes is accompanied by an increase in the prevalence of asymptomatic cardioautonomic neuropathy. This age-related excess risk of cardioautonomic neuropathy is not observed in men. Men and women with type 1 diabetes have opposite associations between circulating androgens and indexes of cardioautonomic function.

*Trial registration* ClinicalTrials.gov Identifier: NCT04950634.

**Supplementary Information:**

The online version contains supplementary material available at 10.1186/s12933-023-01766-y.

## Background

A sex difference characterizes cardiovascular outcomes in patients with type 1 diabetes [1]. Cardiovascular events are more prevalent in men than in women from the general population. However, this sex gap is overridden by the presence of diabetes to an extreme where the relative risk of macrovascular events is greater in women with diabetes than in male patients [1]. The sex-related burden of cardiovascular disease dramatically increases in postmenopausal women with diabetes [1, 2]. The reason underneath this fact is likely multifactorial, with contributions from physiological differences inherent to sex biology such as sex hormones and sex-specific cardiovascular risk factor profiles [1].

The impact of sex on cardiovascular autonomic neuropathy (CAN) as presentation of macrovascular disease is unclear, even though the sexual dimorphic regulation of the autonomic system has been proposed as one of the factors responsible for the larger cardiovascular risk of women with type 1 diabetes when compared with men [1]. Data regarding the interplay between sex and CAN are even scarcer and, in the case of type 1 diabetes, somehow controversial [3]. Previous findings of our group suggested a sex difference in the figures of CAN, establishing a plausible explanation for the sex disparity observed in the cardiovascular disease of patients with type 1 diabetes [4]. Whilst older men showed a similar prevalence of CAN than their younger male counterparts, menopause in women was followed by a dramatic increase in the prevalence of CAN compared to reproductive-aged women [4].

Even more interesting, our prior research also supported the influence of sex steroids on the prevalence of CAN observed in men and women with type 1 diabetes, because circulating total testosterone (T) concentrations and their relation to estradiol (E_2_) showed opposite associations with indexes of cardioautonomic function [4]. However, since the immunoassays used to measure sex hormones in this earlier report lack the sensitivity, specificity and overall accuracy needed to assess circulating T in women with certainty [5], these results remained in need of confirmation using state-of-the-art assays.

To solve these crucial questions, we aimed to confirm the previous results improving the quality of our study by: (i) increasing our sample size recruiting a new cohort of patients to broaden the representation of the female and male population over 50 years of age, which was limited in our earlier report, and (ii) using a gold-standard method such as liquid chromatography–tandem mass spectrometry (LC–MS/MS) to measure circulating sex hormones.

## Methods

### Study population

In this new cross-sectional study conducted from January 2018 to December 2021, we consecutively recruited 345 adults patients with a diagnosis of type 1 diabetes regularly attending our diabetes outpatient clinic of an Academic Hospital from Madrid, Spain (*ClinicalTrials.gov Identifier: NCT04950634*). If some of those consecutive patients had been enrolled in our previous study, they were again recruited after signing a new consent, and all study procedures were again performed regardless the prior ones. The diagnosis of type 1 diabetes required a previous episode of ketoacidosis and/or diabetic autoimmunity, and mandatory use of insulin for survival, following the American Diabetes Association criteria [6]. Exclusion criteria were: (i) age ≥ 85 years; (ii) inability to complete or understand CAN assessment; (iii) clinically evident manifestations of CAN (assessed by the Composite Autonomic Symptom Scale-31; (COMPASS-31) questionnaire) [7]; (iv) diabetic foot; (v) end-stage renal disease or renal replacement therapy; (vi) ongoing pregnancy; and (vii) diagnosis of types of diabetes mellitus other than type 1 diabetes. Age ≥ 85 years was chosen among exclusion criteria because cardiovascular autonomic responses have shown a significant decline with increasing age, and because of age-related values for the expiration to inspiration (E/I) ratio assessed during heart rate (HR) variation with deep breathing do not apply in these individuals [8].

**Fig. 1:**
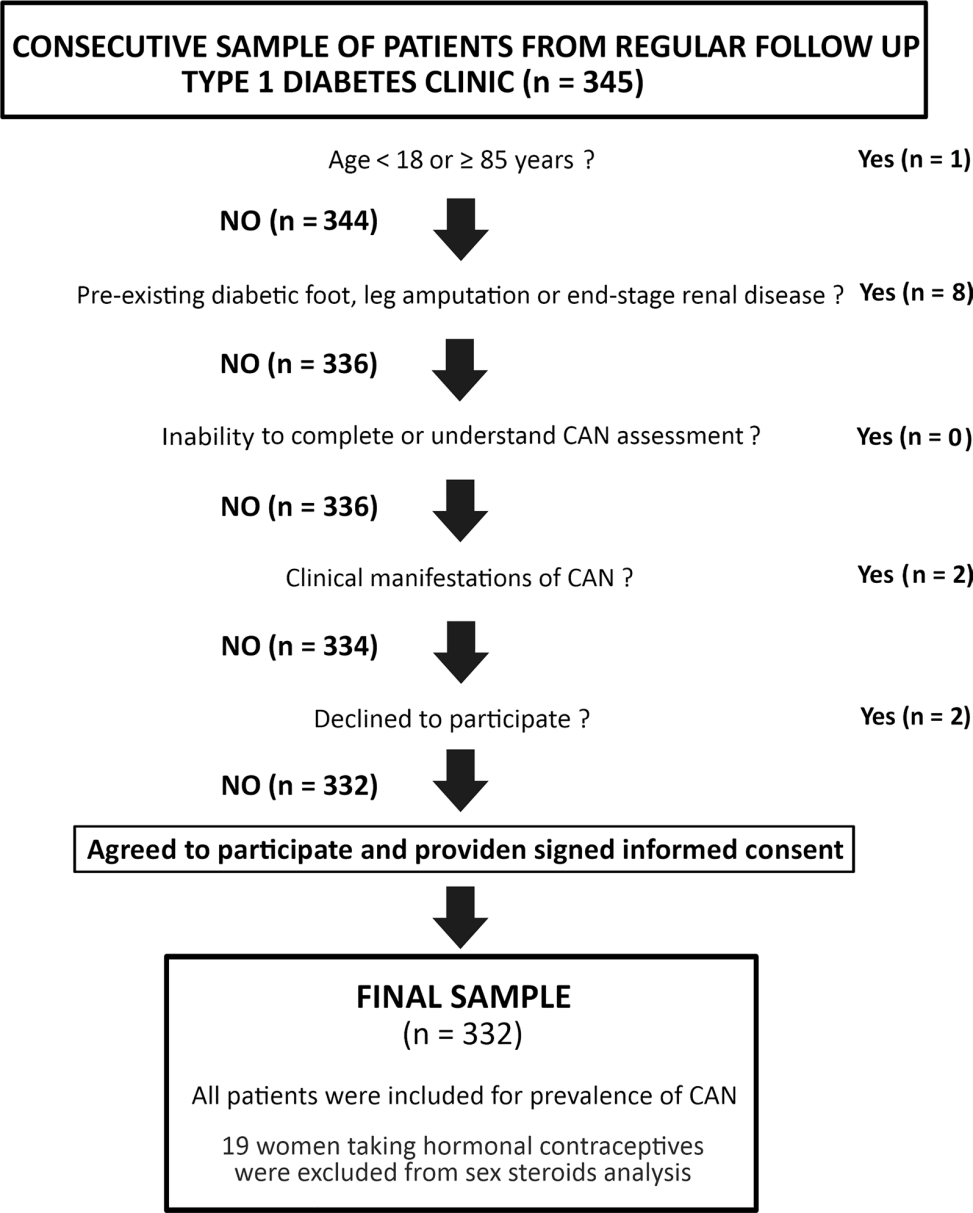
Flow chart of the study

Among the whole group of study subjects, only two patients were excluded due to a diagnosis of severe symptomatic CAN. The participants included into the study, according to exclusion criteria are shown in the Fig. 1.

### Anthropometric, biochemical, and clinical variables

We reviewed the medical records of the patients for their past medical history and medications including clinical parameters related to type 1 diabetes at recruitment. Then, the study subjects were submitted to a complete anthropometric evaluation.

Microvascular complications—including any type 1 diabetes-related eye disease; neuropathy considered as any type 1 diabetes-related neurological complication; and nephropathy considered as any type 1 diabetes-related kidney disease—and macrovascular complications (cerebrovascular disease, coronary artery disease and peripheral arterial disease) were recorded. We also assessed all patients for diabetic peripheral neuropathy [9] by means of detailed clinical history, clinical tests for large-fiber function, protective sensation and detection of feet at risk for ulceration by a 128-Hz tuning fork for vibration perception, ankle reflexes, and a 10-g monofilament test.

An analytical assessment of the renal function, serum lipid profiles, urinary albumin-to-creatinine ratio (UACR), and A_1c_ concentrations were performed at the time of recruitment. Samples for sex steroid measurement were immediately centrifuged, and aliquots of serum and plasma were separated, coded and frozen at − 80 °C until thawed for analysis.

### Assessment of cardiovascular autonomic function: Ewing’s score and Power Spectral heart rate data

Cardiovascular autonomic function (parasympathetic innervation) was assessed by the tests proposed by Ewing et al. [10], and recommended by the American Diabetes Association’s consensus statement on standardized measures for individuals with diabetes [11]. Following recommendations for individuals with diabetes, seemingly asymptomatic CAN was detected using the two currently available gold standard methods [9, 12, 13]: (i) the standardized cardiac autonomic reflex tests (CARTs) described by Ewing et al. in 1970 [10]; and (ii) power spectral HR variability by analyzing beat-to-beat intervals from short-duration electrocardiogram (EKG) recordings. We used a modification of the Ewing’s score to rate the presence of CAN, which scored HR variability to deep breathing, Valsalva’s maneuver, and orthostatism, as well as the response of systolic blood pressure (ΔSBP) to active standing [10]. These responses were categorized as normal (0 points), borderline (0.5 point), or abnormal (one point). A composite score ≥ 1 was considered diagnostic of CAN [10, 14]. We classified CAN as early or mild when the Ewing’s score was between 1 and 2, or as definite when the score was ≥ 2.

Between 7:00 and 9:00 AM, and after resting in supine for at least 10 min in a space with stable temperature, we assessed HR variability using a Monitor VitalScan Medeia^®^ System device (United States, CA). All patients were examined at fasting and not taking their usual medication, except for basal insulin. Participants were instructed to avoid food (nicotine, and caffeine) and particular pharmacological agents (antidepressants, neuroleptics, and antihypertensives) for the 12 h preceding the study procedures. Before obtaining cardiovascular autonomic function studies, we assayed serum glucose in all participants to rule out hypoglycemia. No patient had a serum glucose < 70 mg/dl, which is the glycemic threshold for epinephrine release [15].

The HR response during deep breathing was established by calculating the ratio of the maximum and minimum HR during six cycles of paced deep breathing (E/I ratio). First, the HR response to deep breathing was measured by six deep breathing cycles in 1 min, the maximum and minimum R-R intervals were recorded, and the HR was then calculated. Then, the difference between the maximum and minimum HR was determined. A difference value ≥ 15 indicated normal, 11–14 signified borderline, and ≤ 10 indicated abnormal results. Second, HR response to the Valsalva’s maneuver was assessed by calculating the ratio of the longest R–R interval after the maneuver to the shortest interval during or shortly after the maneuver. The following criteria were used: differences in value ≥ 1.21, 1.11–1.20, ≤ 1.10 indicated normal, borderline, and abnormal results. Moreover, HR response to standing (30:15 ratio) was calculated as the ratio of the longest R-R interval (found at approximately beat 30) to the shortest interval (found at approximately beat 15) after standing up. Differences in value of ≥ 1.04, 1.01–1.03, and ≤ 1.0 signified normal, borderline, and abnormal responses to standing, respectively.

Adrenergic innervation was assessed by the changes in BP and HR 5 min after active standing, from the values recorded while resting in supine. A difference of  ≤ 10 mmHg indicates normal, 11–29 mmHg indicates borderline, and ≥ 30 indicates abnormal results. Orthostatic hypotension was defined by a fall in response to standing > 20 mmHg for systolic BP [9]. HR at resting was measured by palpating the radial pulse and counting the number of beats during 60 s. Resting tachycardia was defined by a HR > 100 beats per minute [9].

We obtained power spectral HR data by analyzing the time series of beat-to-beat intervals from short-duration EKG recordings (10 min) using specialized frequency-domain software VitalScan Medeia^®^ (United States, CA) [8, 12, 16]. This method uses the Fourier method, which transforms R–R intervals into wavelets with two basic components: low Frequency (LF) 0.04–0.15 Hz. and high Frequency (HF) 0.15–0.4 Hz. LF activity represents the combined effects of sympathetic and parasympathetic influence, whereas HF represents parasympathetic activity [8, 16].

### Sex steroid assays

We analyzed total T, sex hormone-binding globulin (SHBG), luteinizing hormone (LH), follicle-stimulating hormone (FSH), and E_2_ in serum samples by personal of laboratory blinded to patient's features and sex. All participants had hormones assayed at 8:00 AM. Serum total T and total E_2_ were measured by LC–MS/MS at the Laboratory of Clinical Biology of the University of Ghent, Belgium, using an AB Sciex 6500 triple-quadrupole mass spectrometer (AB Sciex, Toronto, Canada). The lower limit of quantification (LLOQ) was 1.2 ng/dl (0.04 nmol/l) for total T and the interassay CV was 8.3% at 36.7 ng/dl (1.27 nmol/l) and 3.1% at 307.8 ng/dl (10.68 nmol/l). Serum LLOQ was < 0.5 pg/ml (1.9 pmol/l) for E_2_ and the interassay CV was 4.0% at 21 pg/ml (77 pmol/l).

In all patients, SHBG was assayed by an automated immunochemiluminescence technique (IMMULITE 2000, Siemens Healthcare Sector, Erlangen, Germany) with a LLOQ of 0.02 nmol/l and mean intraassay and interassay CVs < 10%. Calculated free T was assessed by the Vermeulen formula [17], using the ISSAM online calculator (http://www.issam.ch/freetesto.htm). A default albumin level of 4.3 g/dl was used for this calculation. We also calculated free E_2_ levels from their total levels and SHBG concentration.

LH and FSH were measured in a single assay using an automated immunochemiluminescence method (Architect^®^ FSH, Architect^®^ LH, Abbot Ireland diagnostics Division, Lisnamuck, Longford, Co. Longford, Ireland) with LLOQ of 0.1 IU/l. and mean intraassay and interassay CVs < 10% for both assays. To define hypogonadism in males, we use the in-house lower limit of normality for free T (free T less than 225 pmol/l) [18].

### Sample size calculation

We used the online sample size and power calculator provided by the Institut Municipal d'Investigació Mèdica from Barcelona, Spain, version 7.12 (https://www.imim.cat/ofertadeserveis/software-public/granmo/). Assuming a global prevalence of CAN of approximately 30%, as formerly published for our whole population of patients with type 1 diabetes presenting with asymptomatic peripheral artery disease [19], and setting alpha at 0.05 and beta at 0.20 for a two-sided test, the inclusion of at least 180 men and 150 women would be enough to recognize a difference in the prevalence among them greater than 15% by an Arcsinus approximation.

### Statistical analysis

To delineate the influence of the physiologic decline of sex steroids, especially in women during the menopausal transition, we divided our sample of patients in subgroups by age using a cutoff of 50 yr-old, which is the median age at natural menopause in Caucasian women [20]. We also defined menopause according to the Stages of Reproductive Aging Workshop (STRAW) staging system developed from data from multiple longitudinal cohort studies [21], considered the gold standard for characterizing reproductive aging. Women were divided into two groups: (i) women of reproductive age and, (ii) women in late perimenopause (characterized by amenorrhea > 60 days plus a circulating FSH > 25 IU/l) or menopause (defined retrospectively after 12 months of amenorrhea). We show data as means ± SD or median (IQR) according to their distribution, and counts (percentages), besides their 95% confidence interval (CI) (lower limit; upper limit) when appropriate. To ensure normality for parametric tests, we applied logarithmic transformations to all skewed continuous variables. Univariate two-way general linear models (GLM) or binary logistic regression analyses were used to analyze continuous and discrete variables, respectively, considering age group (patients ≤ 50 years and patients > 50 years), sex, and their interaction within a single analysis (adjusted for diabetes duration and A_1c_ levels). The method used to calculate the 95% confidential intervals (CI) for the prevalence of CAN was the Wilson score without continuity correction [22]. The 95% CI for the odds was obtained by taking the CI for the proportion, and then converting those proportions into odds. We calculated the CI for the difference between two proportions by the Newcombe-Wilson method without continuity correction [22]. To assess the main determinants of CAN in men and women, separately, we used stepwise binary logistic regression analyses introducing the presence of CAN as dependent variable, and age group [coded as 0  (≤ 50 years) or 1  (over 50 years)], duration of type 1 diabetes, A_1c_, microvascular complications (coded as 0 = absent and 1 = present), as independent variables, Lastly, the association between sex steroids and Ewing’s autonomic function test score was analyzed by Spearman’s correlation analysis. The level of statistical significance set at a *p* value < 0.05. We used SPSS Statistics 23 (SPSS Inc., Chicago, IL, USA).

## Results

### Characteristics of the patients

The demographics and clinical features of the whole population of patients with type 1 diabetes, and the comparisons between women and men as a function of age are summarized in the Table 1 and Additional file 1: Table S1. There were no sex differences in the form of insulin administration (i.e., use of continuous subcutaneous insulin infusion). Glycemic control at recruitment —as measured by A_1c_– was better in men, without difference between age groups. Men also showed higher waist circumference and higher total insulin daily dose compared with women; however, there were no sex differences in insulin dose when adjusted by weight. There were no sex differences in the pharmacological management except that men were more likely to be under statin therapy. Women showed higher fat mass mean values and HDL-cholesterol concentrations than men (Table 1 and Additional file 1: Table S1). Women over 50 years had a longer duration of diabetes, higher waist circumference and fat mass mean values, and were more likely to be under antiaggregant, statin, and antihypertensive therapies, compared with younger women. In addition, women over 50 showed higher fat mass, HDL-cholesterol, and A_1c_ compared with their similarly aged male counterparts (Table 1 and Additional file 1: Table S1). Women also showed increased markers of subclinical inflammation compared with men (Additional file 1: Table S1).

**Table 1:** Demographic, clinical features and sex steroid profile of the whole group of study participants and as a function of sex and age

Variable	All patients	Women			Men			*P*
	(n = 332)	All (n = 151)	≤ 50 years (n = 108)	> 50 years (n = 43)	All (n = 181)	≤ 50 years (n = 139)	> 50 years (n = 42)	
Age (years)^b^	41 ± 13	41 ± 14	34 ± 10	58 ± 7	40 ± 13	36 ± 10	57 ± 5	** < *0.001***
	(40; 42)	(39; 43)	(32; 36)	(56; 60)	(38; 42)	(34; 38)	(55; 58)	
Age at diagnosis of diabetes (years)^b^	20 ± 11	21 ± 13	17 ± 10	30 ± 15	20 ± 11	17 ± 9	29 ± 12	** < *0.001***
	(19; 21)	(19; 23)	(15; 19)	(26; 34)	(18; 22)	(15; 19)	(25; 33)	
DKA at diagnosis [n (%)]	123 (37)	54 (36)	40 (37)	14 (32)	69 (38)	60 (43)	9 (21)	0.167
	(32; 42)	(29; 44)	(28; 46)	(23; 49)	(31; 45)	(35; 52)	(11; 36)	
CSII [n (%)]	81 (25)	43 (29)	32 (30)	11 (26)	38 (21)	29 (21)	9 (21)	0.684
	(20; 29)	(22; 36)	(22; 39)	(16; 40)	(16; 28)	(15; 28)	(11; 36)	
Total insulin dose (U/day)^a^	43 ± 21	38 ± 17	39 ± 18	34 ± 16	48 ± 22	48 ± 21	50 ± 25	** < *0.001***
	(41; 45)	(35; 41)	(36; 42)	(30; 40)	(45; 51)	(45; 52)	(42; 58)	
Daily insulin dose (U/kg/day)	0.58 ± 0.25	0.58 ± 0.26	0.59 ± 0.27	0.52 ± 0.21	0.59 ± 0.24	0.59 ± 0.23	0.61 ± 0.26	0.255
	(0.55; 0.61)	(0.54; 0.62)	(0.54; 0.64)	(0.46; 0.60)	(0.55; 0.63)	(0.55; 0.63)	(0.53; 0.69)	
Duration of diabetes (years)^b^	19 ± 12	19 ± 12	15 ± 10	26 ± 13	19 ± 11	17 ± 11	27 ± 9	** < *0.001***
	(18; 20)	(17; 21)	(13; 17)	(22; 29)	(17; 21)	(15; 19)	(24; 30)	
Never smokers [n (%)]^b^	193 (59)	88 (58)	70 (65)	18 (42)	105 (58)	87 (63)	18 (43)	** < *0.005***
	(53; 63)	(50; 66)	(55; 73)	(30;58)	(51; 65)	(54; 70)	(29; 58)	
Antiaggregant therapy [n (%)]^b^	41 (12)	15 (10)	3 (3)	12 (28)	26 (14)	7 (5)	19 (45)	** < *0.001***
	(9; 16))	(7; 19)	(1; 8)	(16; 40)	(10; 20)	(3; 10)	(31; 60)	
Statin therapy [n (%)]^a,b^	122 (37)	48 (32)	17 (16)	31 (72)	74 (41)	38 (27)	36 (86)	** < *0.005***
	(32; 42)	(25; 40)	(8; 21)	(60; 84)	(34; 48)	(21; 35)	(72; 93)	
Antihypertensive therapy [n (%)]^a,b,c^	54 (16)	23 (15)	3 (3)	20 (46)	31 (17)	14 (10)	17 (41)	** < *0.005***
	(13; 21)	(10; 22)	(1; 7)	(32; 60)	(12; 23)	(6; 16)	(27; 56)	
Microangiopathy [n (%)]	69 (21)	32 (21)	15 (14)	17 (40)	37 (20)	23 (17)	14 (33)	0.563
	(17; 25)	(15; 28)	(8; 21)	(26; 54)	(15; 27)	(11; 24)	(21; 49)	
Macroangiopathy [n (%)]	18 (5)	7 (5)	0 (0)	7 (16)	11 (6)	3 (2)	8 (19)	0.532
	(4; 8)	(2; 9)	(0; 4)	(8; 28)	(3; 11)	(1; 6)	(9; 33)	
Body mass index (kg/m^2^)	25 ± 4	24 ± 4	24 ± 4	25 ± 5	25 ± 5	25 ± 4	26 ± 3	0.550
	(24; 25)	(23; 25)	(23; 25)	(24; 27)	(24; 26)	(24; 27)	(25; 27)	
Obesity [N (%)]	32 (10)	16 (11)	10 (9)	6 (14)	16 (9)	15 (11)	1 (2)	0.101
	(7; 13)	(7; 17)	(5; 16)	(8; 28)	(6; 14)	(7; 17)	(0; 12)	
Waist circumference (cm)^a,b^	85 ± 13	79± 12	77 ± 12	85 ± 12	89 ± 12	86 ± 11	94 ± 9	** < *0.005***
	(84; 86)	(77; 81)	(76; 80)	(79; 87)	(87; 91)	(84; 88)	(91; 97)	
Fat mass (%)^a,^^b^	24 ± 10	30 ± 8	29 ± 7	33 ± 8	18 ± 8	17 ± 8	22 ± 6	** < *0.001***
	(23; 25)	(29; 31)	(28; 30)	(31; 35)	(17; 19)	(16; 18)	(20; 24)	
eGFR (ml/min/1.73m^2^)^a,b^	90 ± 16	84 ± 16	88 ± 15	77 ± 13	95 ± 15	96 ± 15	88 ± 10	** < *0.001***
	(88; 92)	(81; 87)	(85; 91)	(73; 81)	(93; 97)	(94; 99)	(85; 91)	
A_1c_ (%)^a^	7.2 ± 1.0	7.4 ± 1.1	7.3 ± 1.1	7.6 ± 0.9	7.1 ± 1.0	7.1 ± 1.0	7.1 ± 0.9	** < *0.005***
	(7.1; 7.3)	(7.2; 7.6)	(7.1; 7.5)	(7.3; 7.9)	(6.9; 7.3)	(6.9; 7.3)	(6.8; 7.4)	
A_1c_ (mmol/mol)	56 ± 11	57 ± 12	57 ± 13	59 ± 10	54 ± 11	54 ± 11	54 ± 10	** < *0.005***
	(54; 57)	(55; 59)	(55; 60)	(56; 62)	(52; 56)	(52; 56)	(51; 57)	
UACR (mg/g)	5.8 (4.9)	6.8 (5.8)	6.6 (5.3)	7.8 (8.5)	5.1 (4.4)	5.1 (4.3)	5.1 (4.5)	0.037
FSH (IU/l)^a,b,c^^*^	4 (4)	6 (40)	5 (4)	64 (30)	4 (3)	3 (3)	5 (3)	** < *0.001***
LH (IU/l)^a,b,c^^*^	4 (4)	6 (16)	5 (5)	25 (12)	3 (2)	3 (2)	3 (2)	** < *0.001***
Total T (nmol/l)^a^^*^	13.6 (22.8)	1.1 (0.7)	1.1 (0.8)	0.9 (0.6)	22.6 (11.3)	22.9 (11.8)	22.2 (9.9)	** < *0.001***
Total E_2_ (pmol/l)^a,b,c^^*^	98 (89)	168 (356)	355 (376)	21 (38)	90 (46)	90 (44)	88 (50)	** < *0.001***
Total T/ E_2_ molar ratio^a,b,c^*	166 (250)	9 (22)	3 (5)	36 (53)	251 (125)	256 (129)	232 (107)	** < *0.005***
SHBG (nmol/l)^a^*	72 ± 40	98 ± 44	96 ± 51	102 ± 29	52 ± 22	50 ± 22	60 ± 22	** < *0.001***
	(68; 76)	(91; 105)	(87; 107)	(90; 108)	(49; 55)	(46; 54)	(53; 67)	
Calculated free T (pmol/l)^a,c^*	235 (380)	10 (8)	11 (8)	8 (6)	381 (168)	408 (179)	337 (122)	** < *0.005***
Calculated free E_2_ (pmol/l)^a,b,c^^*^	1.8 (1.5)	2.6 (4.7)	4.6 (4.7)	0.3 (0.5)	1.7 (0.9)	1.7 (0.9)	1.7 (0.7)	** < *0.005***
Calculated free T/E_2_ molar ratio^a,c^^*^	140 (232)	6 (15)	2 (3)	23 (33)	230 (110)	246 (112)	200 (86)	** < *0.001***

Continuous variables are shown as mean ± SD, or median (IQR). Discrete variables are shown as raw numbers (percentage). Figures below those statistics denote confidence intervals. Comparisons among groups were performed by an univariate two-way GLM or binary logistic regression analyses (adjusted for diabetes duration and A_1c_ levels). Bold italics figures denote statistical significance

ACEI, angiotensin-converting enzyme inhibitor; ARB, angiotensin receptor blocker; BP, blood pressure; CSII, continuous subcutaneous insulin infusion; DKA, diabetes ketoacidosis; eGFR, estimated glomerular filtration rate (MDRD-4 formula); E_2_, estradiol; FSH, follicle-stimulating hormone; HDL, high density-lipoprotein; IU, international units; LDL, low density-lipoprotein; LH, luteinizing hormone; SHBG, sex hormone-binding globulin; T, testosterone; U, units; UACR, urinary albumin-to-creatinine ratio

^*^19 women taking hormonal contraceptives were excluded

^a^Significant differences between men and women

^b^Significant differences among older and younger patients independently of sex

^c^Statistically significant interaction between sex and group of age

As expected, men presented with higher total T, free T, total T/E_2_ and free T/E_2_ molar ratios than women, whereas women had higher total E_2_, free E_2_ and SHBG concentrations than men (Table 1). According to STRAW staging system six women were in late perimenopause period, and forty-one women were postmenopausal.

### Influence of sex on the prevalence and severity of cardiovascular autonomic dysfunction

When considering all subjects as a whole, CAN prevalence (define as a Ewing’s score > 1) was not significantly different between women and men [31.8% (24.9; 39.6) *vs*. 24.3% (18.6; 31.1), respectively, *P* = 0.129]. When age was taken into account, the prevalence of CAN was similar in young men and men over 50 years (Table 2). In agreement, the OR of having CAN in men older than 50 years (compared to their younger counterparts) did not increase [OR 1.8 (0.9; 3.8)]. However, menopause resulted in an excess risk of CAN in women. In female individuals over 50 years (n = 43), the prevalence of CAN doubled that of young women [20.4% (13.7; 29.2) *vs.* 45.8% (32.6; 59.7), respectively, *P* = 0.001, Table 2]. The OR of having CAN increased in women older than 50 years compared to their younger counterparts [3.3 (1.6; 6.9)]. The excess risk of CAN was even more evident when STRAW criteria were used. In women with perimenopause or menopause (n = 47), the prevalence of CAN doubled that of younger women [vs. 51% (37; 65) *vs.* 23% (16; 32), respectively, *P* < 0.001], with an OR 3.5 (1.7; 7.2) of having CAN compared with their reproductive-aged counterparts.

**Table 2:** Tests of cardiovascular autonomic function considering all patients with type 1 diabetes as a whole, and as a function of sex and age

	Cardiovascular autonomic indexes							
	All patients	Women			Men			*P*
	(n = 332)	All (n = 151)	≤ 50 years (n = 108)	> 50 years (n = 43)	All (n = 181)	≤ 50 years (n = 139)	> 50 years (n = 42)	
Resting SBP (mmHg)^a, b^	121 ± 13	117 ± 14	114 ± 11	125 ± 16	124 ± 12	121 ± 10	134 ± 12	** < *0.001***
	(120; 122)	(115; 119)	(111; 115)	(122; 131)	(122; 126)	(119; 123)	(130; 138)	
Resting HR (bpm)	72 ± 11	73 ± 10	73 ± 10	71 ± 9	71 ± 11	71 ± 12	71 ± 10	0.380
	(71; 73)	(71; 75)	(72; 76)	(68; 74)	(69; 73)	(69; 73)	(68; 74)	
∆SBP (mmHg)^a,c^	2 (13)	3 (14)	3 (13)	3 (18)	0 (12)	1 (1)	-1 (11)	** < *0.001***
Orthostatic hypotension [n (%)]	6 (2)	3 (2)	3 (3)	0 (0)	3 (2)	1 (1)	2 (5)	0.997
	(1; 4)	(1; 6)	(1; 8)	(0; 8)	(1; 5)	(0; 4)	(1; 16)	
E/I index^b,c^	1.43 ± 0.28	1.42 ± 0.27	1.45 ± 0.25	1.36 ± 0.33	1.42 ± 0.28	1.47 ± 0.29	1.27 ± 0.20	** < *0.005***
	(1.40; 1.46)	(1.38; 1.46)	(1.37; 1.47)	(1.27; 1.46)	(1.38; 1.46)	(1.42; 1.52)	(1.21; 1.33)	
VAL index^b^	1.39 ± 0.26	1.36 ± 0.30	1.38 ± 0.25	1.30 ± 0.40	1.41 ± 0.22	1.43 ± 0.22	1.34 ± 0.22	** < *0.005***
	(1.36; 1.42)	(1.31; 1.41)	(1.33; 1.43)	(1.18; 1.42)	(1.38; 1.44)	(1.29; 1.47)	(1.27; 1.41)	
30:15 index^a,c^	1.38 ± 0.32	1.34 ± 0.29	1.38 ± 0.27	1.25 ± 0.31	1.41 ± 0.34	1.39 ± 0.33	1.45 ± 0.38	** < *0.005***
	(1.35; 1.42)	(1.29; 1.39)	(1.33; 1.43)	(1.16; 1.34)	(1.36; 1.46)	(1.33; 1.45)	(1.33; 1.57)	
Low-Frequency (LF)^b^	1.9 (1.3)	1.9 (1.4)	2.2 (1.2)	1.1 (1.2)	1.93 (1.28)	2.04 (1.39)	1.25 (1.03)	** < *0.001***
High-Frequency (HF)^b^	1.8 (1.7)	2.1 (1.9)	2.4 (1.8)	1.1 (1.2)	1.74 (1.70)	1.92 (1.61)	1.10 (1.09)	** < *0.001***
Total Ewing score^a,b^	0.59 ± 0.52	0.68 ± 0.59	0.62 ± 0.59	0.83 ± 0.60	0.52 ± 0.44	0.47 ± 0.44	0.68 ± 0.41	** < *0.050***
	(0.53; 0.65)	(0.59; 0.77)	(0.50; 0.72)	(0.65; 1.01)	(0.46; 0.58)	(0.40; 0.54)	(0.55; 0.81)	
Prevalence of CAN [n (%)]^b^	92 (28)	48 (32)	21 (20)	22 (46)	44 (24)	30 (22)	14 (33)	** < *0.050***
	(23; 33)	(25; 40)	(14; 29)	(33; 60)	(19; 31)	(16; 29)	(21; 49)	

Continuous variables are shown as mean ± SD, or median (IQR). Discrete variables are shown as raw numbers (percentage). Figures below those statistics denote confidence intervals (lower limit; upper limit). Bold italics figures denote statistical significance

CAN was determined by the Ewing’s score (composite score ≥ 1). Comparisons among groups were performed by univariate two-way GLM or binary logistic regression analyses (adjusted for diabetes duration and A_1c_ levels). Nineteen women taking hormonal contraceptives were not included into these analyses

CAN, cardiovascular autonomic neuropathy; DBP, diastolic blood pressure; E/I index, expiration/inspiration index; HR, heart rate; ∆SBP, response in systolic blood pressure to orthostatism; VAL index, Valsalva index

^**a**^Significant differences between men and women

^**b**^Significant differences among older and younger patients independently of sex

^**c**^Statistically significant interaction between sex and group of age

**Fig. 2:**
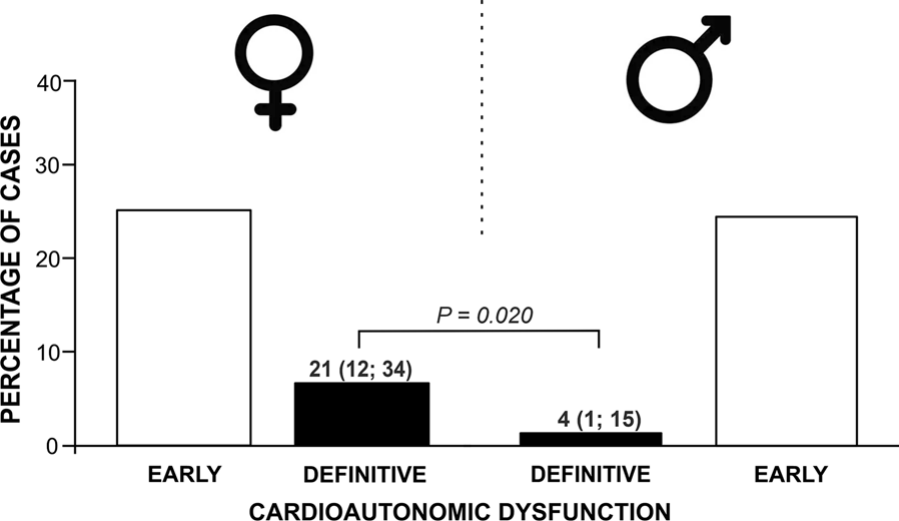
Severity of cardioautonomic dysfunction as a function of sex. The figures above the bars show percentages (%) and 95% confidence intervals (lower limit; upper limit)

Furthermore, women with CAN presented more severe cardioautonomic dysfunction compared with men (Fig. 2). CAN was categorized as early/mild in 38 cases [79% (66; 88)] and definitive in 10 [21% (12; 34)] in women, whereas in men these findings were definitive in only 2 cases [4% (1; 15)] (Fig. 2; *P* = 0.020). The OR of having definitive CAN (defined by a Ewing’s score ≥ 2) was 5.5 (1.2; 26.8) in women compared with men.

Orthostatic hypotension was present in 3 [2% (0.7; 6.0)] female patients and in 3 [2% (0.6; 5.0)] men. Finally, only 1 woman [1% (0.7; 3.8)] and 3 men [2% (0.6; 5.0)] had resting tachycardia.

In women, the binary logistic full regression model showed an age over 50 years and a previous microvascular complication as statistically significant determinants of CAN (Table 3). In men, however, the full model displayed the presence of any other microvascular complication as only significant determinant of CAN (Table 3).

**Table 3:** Main determinants of cardioautomic neuropathy as a function of sex

Dependent variable: CAN diagnosis				
Independent variables	ß	95% CI		*P*
		Lower	Upper	
Full factorial model (Nagelkerke’s R^2^ = 0.161; *P* = 0.001)				
Women				
Age group > 50 years	2.749	1.176	6.422	***0.020***
Any microvascular complication	2.608	1.027	6.622	***0.044***
Duration of diabetes	0.990	0.955	1.026	0.587
Metabolic control	1.409	0.992	2.001	0.055
Full factorial model (Nagelkerke’s R^2^ = 0.127; *P* = 0.004)				
Men				
Age group > 50 years	1.498	0.638	3.516	0.354
Any microvascular complication	2.676	1.105	6.478	***0.029***
Duration of diabetes	1.021	0.983	1.060	0.289
Metabolic control	1.343	0.951	1.897	0.094

The main determinants of cardioautonomic neuropathy in men and women by separate were addressed by binary logistic regression analyses. Bold italics figures denote statistical significance

Independent variables were introduced as follows: age group (≤ 50 years = 0, > 50 years = 1), duration of type 1 diabetes (years), metabolic control (A_1c),_ and any microvascular complication (absent = 0, present = 1)

### Association of sex steroids with tests of cardiovascular autonomic function

**Fig. 3:**
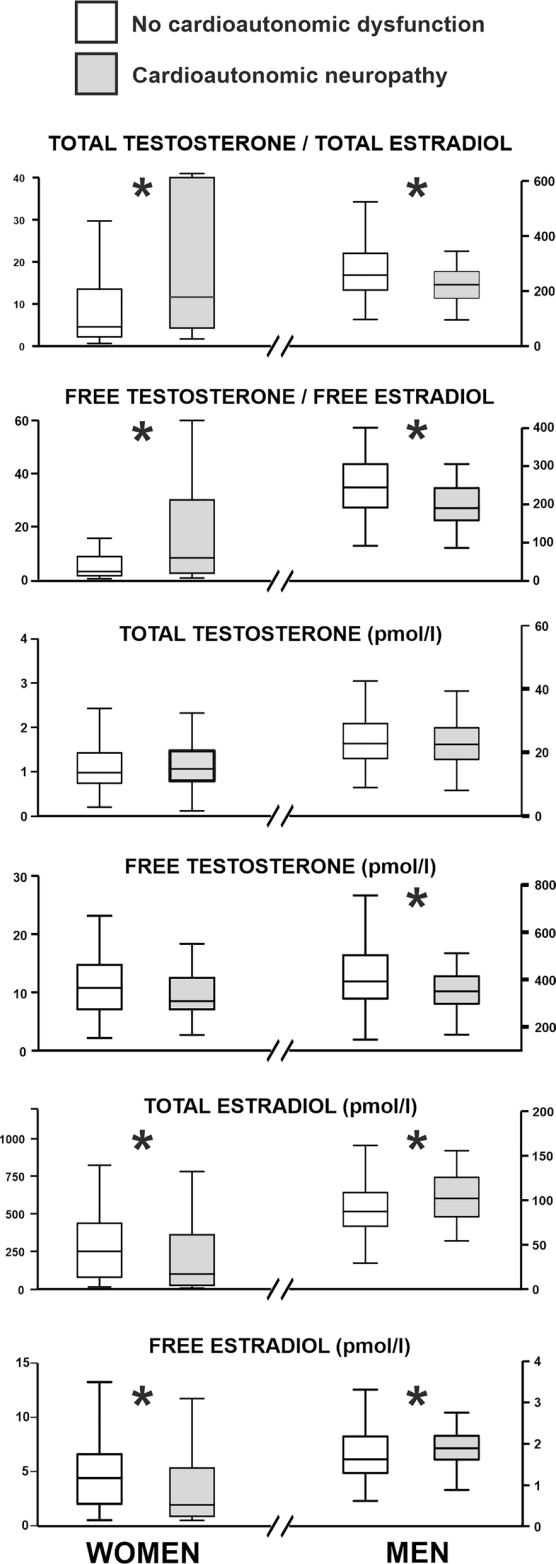
Mean basal steroids levels in women and men with T1D as a function of the presence or not of cardioautonomic neuropathy. The box-plot includes the median (solid horizontal line), and the interquartile range (box), and the whiskers indicate the minimum and maximum data values, unless outliers are present in which case the whiskers extend to a maximum of 1.5 times the inter-quartile range. White boxes represent those patients without a diagnosis of CAN, whereas the values of patients with CAN as showed in grey boxes. *P < 0.05 for the differences between patients with CAN and without CAN

Circulating basal levels of sex steroids are displayed in the Fig. 3. Type 1 diabetic men with CAN showed lower free T, total T/E_2_, free T/E_2_ molar ratios, and higher total and free E_2_ compared with those without CAN. Three out of 13 [23% (8; 50)] male participants with hypoandrogenemia also had CAN, although the prevalence among normonandrogenemic male patients was similar [44 out of 181, (24% (19; 31))].

Conversely, women with type 1 diabetes and CAN had higher total T/E_2_ and free T/E_2_ molar ratios, lower E_2_, and free E_2_, compared with those without CAN (Fig. 3). Sex steroid profiles as a function of sex, age and CAN status are showed in the Additional file 1: Table S2. In correlation analyses, several individual cardioautonomic test scores directly correlated with circulating total T/E_2_ and free T/E_2_ molar ratios in men, and indirectly in women. Nonetheless, correlations with total Ewing’s scores only reached statistical significance in men and young women (Table 4 and Additional file 1: Table S3).

**Table 4:** Correlations between sex steroids and autonomic function tests in men and women with type 1 diabetes

CARTs	Total T	Total E_2_	Total T/E_2_	Free T	Free E_2_	Free T/E_2_
Men						
E/I ratio	**0.169**	− 0.038	**0.217**	**0.257**	− 0.015	**0.286**
Valsalva test	0.052	− 0.077	**0.159**	**0.195**	− 0.034	**0.266**
Orthostatism test	0.031	**0.151**	0.088	0.128	− 0.187	− 0.045
∆SBP	0.007	0.055	− 0.096	0.106	0.107	− 0.034
Total Ewing score	− 0.091	0.141	**− 0.229**	**− 0.214**	0.083	**− 0.282**
HFa	0.104	0.035	0.089	**0.197**	0.067	0.146
LFa	0.110	0.024	0.094	**0.195**	0.050	0.131
Women						
E/I ratio	0.056	0.175	− 0.174	0.033	0.139	− 0.169
Valsalva test	− 0.002	**0.194**	**− 0.216**	0.168	**0.232**	**− 0.217**
Orthostatism test	0.107	**0.294**	**− 0.274**	0.263	**0.297**	**− 0.229**
∆SBP	− 0.033	**0.193**	**− 0.194**	**0.216**	− 0.143	**0.227**
Total Ewing score	0.082	− 0.105	0.136	0.105	− 0.103	0.104
HFa	0.023	**0.308**	**− 0.332**	0.161	**0.313**	**− 0.302**
LFa	0.099	**0.321**	**− 0.324**	0.161	**0.315**	**− 0.306**

Data are Spearman’s rho coefficient of correlation

Data on bold-face denotes statistically significant correlations (*P* < 0.050)

Nineteen women taking hormonal contraceptives were not included into these analyses

## Discussion

Our results confirm that the prevalence of seemingly asymptomatic CAN is markedly increased in female patients with type 1 diabetes age 50 and older. Whilst older men have a similar prevalence of asymptomatic CAN than their younger male counterparts, menopause in women is followed by a dramatic increase in the prevalence of CAN compared to reproductive-aged women in whom the odds of having CAN was 3.5 times greater than in young women patients. This excess risk of CAN might contribute to explain the marked increase in the incidence of cardiovascular disease that happens following the menopausal transition in women with type 1 diabetes. Our findings would also suggest that sex steroids might be involved in the etiopathogenesis of CAN, even though further studies need to be designed for confirmation. Circulating T and their relation to E_2_ appears to have opposite associations with the cardioautonomic regulation of men and women with type 1 diabetes. Women with CAN had lower E_2_ and free E_2_ concentrations, as well higher T/E_2_ ratios, compared with female patients without CAN, whereas men with CAN presented with lower free T concentrations and T/E_2_ ratios in comparison with male patients without autonomic disease. This association was evident in both men and women with type 1 diabetes, who showed positive and negative correlations, respectively, between T concentrations and cardioautonomic function scores.

CAN is a common complication of type 1 diabetes that associates increased morbidity and mortality [23], even in apparently asymptomatic stages [24]. In the DCCT/EDIC cohort, individuals diagnosed with CAN at DCCT closeout experienced a higher long-term risk of cardiovascular events during their follow-up in the EDIC [9]. The EURODIAB Prospective Complications Study showed that CAN is a main predictor for mortality during a 7-yr follow-up, even exceeding [25] the deleterious effect of traditional cardiovascular risk factors [26].

The age and duration of disease are two strong risk factors for CAN in patients with type 1 diabetes [9, 27]. Besides these factors, evidence derived from the general population suggests sex differences in the physiologic regulation of the autonomic nervous system [28]. Middle-aged healthy women have a predominant parasympathetic drive and lower sympathetic activity than men [25], even though autonomic function tends to equalize among sexes with ageing [29, 30]. In consonance, women in their postmenopausal years show a direct relationship between markers of whole body sympathetic tone and vascular resistance [25].

Regarding type 1 diabetes, data on the relationship between sex and CAN are scarce and, somehow, controversial [3, 23, 31]. Our previous findings suggest that, after stratifying by age, women over age 45 showed a marked increase in the risk of suffering from CAN, both when compared to younger women, and to men [4]. Our current findings confirm that the prevalence of asymptomatic CAN is similar in both sexes when considering patients with type 1 diabetes as a whole (at least, any possible difference in prevalence is below the 15% established by our power analysis calculations). However, after stratifying by age in order to account for the impact of the menopausal transition in women, those aged 50 years and older showed a marked and statistically significant increased risk of CAN compared with younger women.

This finding led us to question to what extent the evident differences in circulating sex steroids between women and men influenced the prevalence of CAN. Even though we cannot rule out an effect of sex chromosomes and genes on cardiovascular autonomic regulation, a direct influence of sex hormones may underlie these findings. In fact, the modulation of the autonomic system by sex steroids has been proposed to mediate the increase of parasympathetic tone in women [32]. After menopause, normotensive women have a significant reduction in HR variability, which is largely attributed to deprivation from ovarian hormones [33]. Estrogens might be the main hormones at play, since experimental and clinical studies have shown that they pose an important role in autonomic regulation by increasing vagal autonomic modulation, and reducing cardiac sympathetic autonomic modulation [33].

The higher concentrations of total T/E_2_ molar ratio and free T/E_2_ molar ratio in type 1 diabetes women with asymptomatic CAN suggest that androgen excess may be associated with autonomic dysfunction in these women, as has already been reported in euglycemic women with polycystic ovary syndrome [34]. The differences in cardiac autonomic modulation between sexes can be exemplified by those women, in whom the increase in androgens correlated with sympathetic autonomic activity [34]. Hence, T levels in men and estrogens in women are variables that could explain the sex differences found in the autonomic modulation of HR variability. Moreover, exposure of the ovary and adrenals to the exogenous hyperinsulinism that results from the supraphysiological subcutanous insulin doses needed to control gluconeogenesis at the liver, may increase the prevalence of this androgen excess syndrome among women with type 1 diabetes [35].

However, the T/E_2_ molar ratio increase observed in our postmenopausal women largely relied on the marked decrease in E_2_ levels. Supporting the deleterious role of estrogen deficiency on autonomic dysfunction, sex-specific differences in autonomic nervous function —with estrogens facilitating the cardiac parasympathetic nervous control— have been previously reported both in animal models and in healthy human beings [32].

It should be noted that the group of women over age 50 years in our study was similar, in terms of classic cardiovascular risk factors, to the other study subgroups: no differences were found in the prescription of antiplatelet, statin, and antihypertensive drugs compared to men over 50 years of age. Furthermore, our older women also had a longer duration of diabetes —as expected from the inclusion criteria of our study— which is one of the most important predictors of cardiovascular outcomes in type 1 diabetes [36]. However, this non-modifiable factor could only explain our results partially, because the subset of older men with type 1 diabetes in our series, who also had a long duration of diabetes, did not show a higher prevalence of CAN than that of younger male patients.

Interestingly, T concentrations were positively associated with HR variability in men with type 1 diabetes, and lower T levels were observed among those diagnosed of asymptomatic CAN. In contrast to women, low T levels in men from the general population are associated with atherosclerosis, coronary artery disease, and cardiovascular events [37]. Moreover, low T concentrations associate with autonomic dysfunction in a few studies conducted in men with known cardiovascular disease [38, 39]. Anyhow, men with type 1 diabetes do not appear to have an increased prevalence of androgen deficiency with respect to the general population [40], and the only study assessing this issue in type 1 diabetes demonstrated weak associations with continuous markers of CAN such as the Valsalva test score [1]. In keeping with these findings, we also found direct associations between T concentrations and cardiovascular function test scores.

We realize that our study is not free of limitations: (i) the cross-sectional and observational design of our study precludes inferring any causality to the association between sex steroid levels and autonomic dysfunction; (ii) despite implementing a rigorous statistical analysis adjusting for multiple comparisons when possible, multiplicity might lead to spurious associations in some cases (type 1 error); (iii) patients with evident clinical manifestations of CAN were excluded from the present analysis, and therefore, we cannot rule out a selection bias; however, only 2 subjects were excluded from the study because of this reason; (iv) we included 19 women taking hormonal contraceptives in our analyses of asymptomatic CAN prevalence. The effect of hormonal contraceptives on sympathetic nerve activity is unclear in healthy women, although the nocturnal fall in BP, which is sympathetically mediated, might be affected by its use [41]; (v) we obtained our serum samples in premenopausal women regardless of the phase of their menstrual cycle, and thus we were not able to assess this source of physiologic variability in sex steroid concentrations. However, the impact of menstrual variability on androgen concentrations is small. Furthermore, the main hypothesis of our work focuses on the dramatic increase in the prevalence of cardioautonomic dysfunction in women, especially after menopause, contributing to the already widely demonstrated increase in cardiovascular morbidity of this stage of life in women. Consequently, although the determination of sex steroids was random, this physiological variability is not present in postmenopausal women (the most interesting group for the study), nor in men. Thus, we believe that this limitation in the study design does not excessively affect the primary objective set out on the basis of our hypothesis.

## Conclusions

We consider fulfilled our aim of improving our previous report [4], because the representation of patients over 50 years of age (women and men) with similar baseline characteristics is much larger, we have assayed sex steroids with a gold standard technique, and performed an exhaustive exploration of the autonomic cardiovascular system. Our current data confirm and expands novel evidence about the presence of sex differences in the prevalence of asymptomatic CAN in type 1 diabetes, with menopause in women playing a major role. While the prevalence of apparently asymptomatic CAN is similar in men with type 1 diabetes across age ranges, menopausal status in women with type 1 diabetes is accompanied by a dramatic increase in its prevalence. Given the association of CAN with cardiovascular disease, the high prevalence of asymptomatic autonomic dysfunction among women with type 1 diabetes older than 50 years suggests the need to assess screening programs for CAN in postmenopausal women with the aim of improving their cardiovascular risk stratification. Finally, since certain normality thresholds used to assess cardioautonomic function vary according to age (the E/I ratio) but not to sex, a question raised by our findings is whether or not those thresholds need to be also different as a function of this latter, a point that is currently unresolved.

## Supplementary Information

**Additional file 1: Table S1**. Selected variables from study population including microangiopathy subtype, antihypertensive therapy, markers of subclinical inflammation, and lipid profiles. **Table S2.** Sex steroid profile as a function of sex, age, and CAN status. **Table S3.** Correlations among sex steroids and autonomic function tests in men and women stratified by age.

## Data Availability

All data sets generated during and/or analyzed during the current study are not publicly available but are available from the corresponding author on reasonable request.

## Abbreviations

BP: Blood pressure
CAN: Cardioautonomic neuropathy
CARTs: Cardiac autonomic reflex tests
CI: Confidence interval
CSII: Continuous subcutaneous insulin infusion
CV: Coefficient of variation
E_2_: Estradiol
EKG: Electrocardiogram
E/I: Expiration to inspiration ratio
ESR: Erythrocyte sedimentation rate
FSH: Follicle stimulating hormone
GLM: General linear model
HF: High frequency
HR: Heart rate
hs-CRP: High sensitivity-C reactive protein
LC–MS/MS: Liquid chromatography–tandem mass spectrometry
LF: Low frequency
LLOQ: Lower limit of quantification
LH: Luteinizing hormone
PAD: Peripheral artery disease
T: Testosterone
∆SBP: Systolic blood pressure response to active standing
SHBG: Sex hormone-binding globulin
UACR: Urinary albumin-to-creatinine ratio
