# Supplementary material for: Sex differences and sex steroids influence on the presentation and severity of cardiovascular autonomic neuropathy of patients with type 1 diabetes
Source: Cardiovasc Diabetol. 2023 Feb 15;22:32. doi: 10.1186/s12933-023-01766-y (PMC10127589; doi:10.1186/s12933-023-01766-y)
Supplement: Supplementary file 1 — Additional file 1: Table S1. Selected variables from study population including microangiopathy subtype, antihypertensive therapy, markers of subclinical inflammation, and lipid profiles. Table S2. Sex steroid profile as a function of sex, age, and CAN status. Table S3. Correlations among sex steroids and autonomic function tests in men and women stratified by age. [file 12933_2023_1766_MOESM1_ESM.docx]

**Table S1**. Selected variables from study population including microangiopathy subtype, antihypertensive therapy, markers of subclinical inflammation, and lipid profiles.

|  | **All patients** | | | **Women** | | | | | | | | | **Men** | | | | | | | | |
| --- | --- | --- | --- | --- | --- | --- | --- | --- | --- | --- | --- | --- | --- | --- | --- | --- | --- | --- | --- | --- | --- |
|  | (n = 332) | | | All  (n = 151) | | | ≤ 50 yrs  (n = 108) | | | > 50 yrs  (n = 43) | | | All  (n = 181) | | | ≤ 50 yrs  (n = 139) | | | > 50 yrs  (n = 42) | | |
| Microangiopathy [n (%)] | 69 (21) | | | 32 (21) | | | 15 (14) | | | 17 (40) | | | 37 (20) | | | 23 (17) | | | 14 (33) | | |
|  | (17; 25) | | | (15; 28) | | | (8; 21) | | | (26; 54) | | | (15; 27) | | | (11; 24) | | | (21; 49) | | |
| -Retinopathy [n (%)] | 46 (14) | | | 21 (14) | | | 11 (10) | | | 10 (23) | | | 25 (14) | | | 16 (12) | | | 9 (21) | | |
|  | (11; 18) | | | (9; 20) | | | (5; 17) | | | (14; 38) | | | (10; 20) | | | (7; 18) | | | (12; 36) | | |
| -Nephropathy [n (%)] | 24 (7) | | | 11 (7) | | | 7 (7) | | | 4 (9) | | | 13 (7) | | | 8 (6) | | | 5 (12) | | |
|  | (5; 11) | | | (4; 13) | | | (3; 13) | | | (3; 20) | | | (4; 12) | | | (3; 11) | | | (5; 25) | | |
| -Peripheral neuropathy [n (%)] *^b^* | 28 (8) | | | 14 (9) | | | 3 (3) | | | 11 (26) | | | 14 (8) | | | 8 (6) | | | 6 (14) | | |
|  |  | | |  | | |  | | |  | | |  | | |  | | |  | | |
| Antihypertensive therapy [n (%)] ^a,^ ***^b, c^*** | 54 (16) | | | 23 (15) | | | 3 (3) | | | 20 (47) | | | 31 (17) | | | 14 (10) | | | 17 (41) | | |
|  | (13; 21) | | | (10; 22) | | | (1; 7) | | | (32; 60) | | | (12; 23) | | | (6; 16) | | | (27; 56) | | |
| ACEI or ARB, n (%) | 35 (65) | | | 13 (57) | | | 3 (100) | | | 10 (52) | | | 13 (42) | | | 10 (71) | | | 12 (71) | | |
|  | (52; 76) | | | (37; 74) | | | (34; 100) | | | (32; 72) | | | (29;52) | | | (45; 88) | | | (47; 87) | | |
| Diuretics, n (%) | 2 (4) | | | 2 (9) | | | 0 (0) | | | 2 (10) | | | 2 (9) | | | 0 (0) | | | 0 (0) | | |
|  | (1; 12) | | | (2; 27) | | | (0; 66) | | | (3; 29) | | | (4; 42) | | | (0; 22) | | | (0; 18) | | |
| β-blockers, n (%) | 8 (15) | | | 3 (13) | | | 0 (0) | | | 3 (14) | | | 5 (16) | | | 2 (14) | | | 3 (18) | | |
|  | (8; 27) | | | (5; 32) | | | (0; 66) | | | (5; 35) | | | (2; 64) | | | (4; 40) | | | (6; 41) | | |
| ACEI or ARB + diuretics, n (%) | 11 (20) | | | 6 (26) | | | 0 (0) | | | 6 (29) | | | 5 16) | | | 2 (14) | | | 3 (18) | | |
|  | (12; 33) | | | (13; 47) | | | (0; 66) | | | (14; 50) | | | (2; 64) | | | (4; 40) | | | (6; 41) | | |
| ACEI or ARB + calcium channel blockers, n (%) | 6 (11) | | | 2 (9) | | | 0 (0) | | | 2 (10) | | | 4 (13) | | | 2 (14) | | | 2 (12) | | |
|  | (5; 22) | | | (2; 27) | | | (0; 66) | | | (3; 29) | | | (5; 29) | | | (4; 40) | | | (3; 34) | | |
| -Monotherapy | 32 (59) | | | 12 (52) | | | 3 (100) | | | 9 (48) | | | 20 (65) | | | 10 (71) | | | 10 (59) | | |
|  | (46; 71) | | | (33; 71) | | | (34; 100) | | | (28; 68) | | | (47; 79) | | | (45; 88) | | | (36; 78) | | |
| -Dual therapy | 16 (30) | | | 9 (39) | | | 0 (0) | | | 9 (43) | | | 7 (23) | | | 2 (14) | | | 5 (29) | | |
|  | (19; 43) | | | (22; 59) | | | (0; 66) | | | (24; 63) | | | (11; 40) | | | (4; 40) | | | (13; 53) | | |
| -Triple therapy | 6 (11) | | | 2 (9) | | | 0 (0) | | | 2 (10) | | | 4 (13) | | | 2 (14) | | | 2 (12) | | |
|  | (5; 22) | | | (2; 27) | | | (0; 66) | | | (3; 29) | | | (5; 29) | | | (4; 40) | | | (3; 34) | | |
| Once /twice daily doses | 35(65)/19(35) | | | 15(65)/8(35) | | | 2(75)/1(25) | | | 13(67)/7(33) | | | 20(64)/11(36) | | | 9(74)/5(36) | | | 11(65)/6(35) | | |
|  | (52; 76)/(24; 49) | | | (45; 81)/(19; 55) | | | (1; 91)/(1; 91) | | | (45; 83)/(17; 55) | | | (47; 79)/(21; 53) | | | (39; 84)/(16; 61) | | | (41; 83)/(17; 59) | | |
| Total cholesterol (mmol/l) ***^a^*** | 4.6 | ± | 2.8 | 4.7 | ± | 0.8 | 4.6 | ± | 0.8 | 4.8 | ± | 0.9 | 4.3 | ± | 1.0 | 4.3 | ± | 0.8 | 4.3 | ± | 1.0 |
| HDL-cholesterol (mmol/l) ***^a, b^*** | 1.6 | ± | 0.4 | 1.7 | ± | 0.4 | 1.7 | ± | 0.4 | 1.8 | ± | 0.5 | 1.4 | ± | 0.3 | 1.4 | ± | 0.3 | 1.5 | ± | 0.3 |
| LDL-cholesterol (mmol/l) | 2.6 | ± | 0.7 | 2.6 | ± | 0.7 | 2.6 | ± | 0.7 | 2.6 | ± | 0.7 | 2.5 | ± | 0.6 | 2.6 | ± | 0.6 | 2.4 | ± | 0.6 |
| Triglycerides (mmol/l) | 0.7 (0.3) | | | 0.6 (0.3) | | | 0.6 (0.3) | | | 0.7 (0.5) | | | 0.7 (0.3) | | | 0.7 (0.3) | | | 0.7 (0.2) | | |
| hs-C reactive protein (mg/l) ***^a^*** | 0.9 (1.8) | | | 1.1 (2.1) | | | 1.0 (2.1) | | | 1.3 (2.0) | | | 0.8 (1.6) | | | 0.8 (1.6) | | | 0.8 (1.7) | | |
| ESR (mm/h)***^a^*** | 4 (4) | | | 6 (7) | | | 6 (6) | | | 7 (9) | | | 3 (2) | | | 3 (2) | | | 4 (2) | | |
| Homocysteine (µmol/l) ***^a^*** | 10 | ± | 4 | 9 | ± | 3 | 9 | ± | 3 | 10 | ± | 3 | 11 | ± | 4 | 11 | ± | 4 | 11 | ± | 3 |
| Fibrinogen (mg/dl) ***^a, b^*** | 299 | ± | 78 | 323 | ± | 83 | 315 | ± | 82 | 342 | ± | 86 | 281 | ± | 68 | 276 | ± | 65 | 298 | ± | 76 |

Data are means ± SD or median (IQR). Comparisons among groups were performed by univariate two-way GLM or binary logistic regression analyses. **^a^** Significant differences between men and women; **^b^** Significant differences among older and younger patients independently of sex; **^c^** Statistically significant interaction between sex and group of age.

**Table S2.** Sex steroid profile as a function of sex, age, and CAN status.

|  | **Women** | | | | **Men** | | | |  |
| --- | --- | --- | --- | --- | --- | --- | --- | --- | --- |
|  | ≤ 50 yrs | | > 50 yrs | | ≤ 50 yrs | | > 50 yrs | | ***P*** |
|  | **Cardioautonomic neuropathy** | | | | | | | |  |
|  | **No**  (n = 82) | **Yes**  (n = 26) | **No**  (n = 21) | **Yes**  (n = 22) | **No**  (n = 110) | **Yes**  (n = 29) | **No**  (n = 27) | **Yes**  (n = 15) |  |
| Age (yrs) ***^b, c #^*** | 34 ± 10 | 35 ± 10 | 58 ± 7 | 59 ± 7 | 35 ± 10 | 40 ± 9 | 56 ± 4 | 58 ± 6 | *<0.005* |
| Duration of diabetes (yrs) ***^b^*** | 15 ± 10 | 16 ± 7 | 27 ± 12 | 27 ± 14 | 16 ± 11 | 22 ± 11 | 27 ± 7 | 27 ± 10 | *<0.001* |
| A_1c_ (%) ***^c^*** | 7.2 ± 1.1 | 7.6 ± 1.2 | 7.4 ± 0.9 | 7.8 ± 0.9 | 7.0 ± 1.0 | 7.4 ± 0.9 | 7.0 ± 0.9 | 7.3 ± 0.9 | *<0.005* |
| Total T (nmol/l)*** ***^a^*** | 1.1 (0.7) | 1.5 (0.9) | 0.9 (0.8) | 1.0 (0.6) | 23 (13) | 21 (9) | 21 (9) | 23 (16) | *<0.001* |
| Total E_2_ (pmol/l)*** ***^a, b, d^*** | 279 (319) | 357 (550) | 24 (37) | 20 (37) | 90 (37) | 102 (53) | 86 (48) | 102 (42) | *<0.001* |
| Total T/ E_2_ molar ratio*** ***^a, b, d, e#^*** | 4 (7) | 5 (11) | 35 (37) | 50 (79) | 261 (133) | 223 (97) | 235 (118) | 222 (128) | *<0.005* |
| Free T (pmol/l)*** ***^a, d^*** | 11 (9) | 10 (7) | 7 (9) | 8 (5) | 417 (203) | 380 (125) | 337 (139) | 317 (94) | *<0.001* |
| Free E_2_ (pmol/l)*** ***^a, b, d^*** | 4.1 (4.0) | 4.0 (5.5) | 0.4 (0.5) | 0.3 (0.5) | 1.7 (0.9) | 1.9 (0.7) | 1.6 (0.8) | 1.9 (0.4) | *<0.005* |
| Free T/E_2_ molar ratio******^a, c, d, e^*** | 2.4 (4.9) | 2.5 (9.0) | 23 (24) | 29 (45) | 253 (116) | 188 (89) | 206 (99) | 192 (103) | *<0.001* |

Continuous variables are shown as mean ± SD, or median (IQR). CAN was determined by the Ewing’s score (composite score ≥ 1). Comparisons among groups were performed by univariate two-way GLM (adjusted for diabetes duration and A1c levels). Twenty-nine women taking hormonal contraceptives were not included into these analyses.

a Significant differences between men and women.

b Significant differences among older and younger patients independently of sex.

c Significant differences between CAN status (No/Yes).

d Statistically significant interaction between sex and group of age.

e Statistically significant interaction between sex and CAN status.

f Statistically significant interaction between sex, group of age, and CAN status.

# Statistical significance disappeared after adjustment for confounding variables (diabetes duration and A1c levels).

**Table S3**: Correlations among sex steroids and autonomic function tests in men and women stratified by age

| **CARTs** | **Total T** | **Total E_2_** | **Total**  **T/E_2_** | **Free T** | **Free E_2_** | **Free**  **T/E_2_** |
| --- | --- | --- | --- | --- | --- | --- |
| **Men** |  |  | **≤ 50 yrs** | |  |  |
| *E/I ratio* | **0.202** | -0.045 | 0.171 | **0.235** | 0.045 | **0.239** |
| *Valsalva test* | 0.093 | -0.052 | **0.175** | **0.180** | -0.052 | **0.264** |
| *Orthostatism test* | 0.041 | -0.145 | 0.088 | 0.141 | 0.145 | -0.047 |
| *∆BP* | 0.011 | 0.096 | -0.138 | 0.068 | 0.096 | -0.092 |
| *Total Ewing score* | -0.133 | 0.061 | **-0.200** | **-0.209** | 0.061 | **-0.251** |
| *HFa* | 0.092 | -0.026 | 0.074 | **0.176** | 0.026 | 0.128 |
| *LFa* | 0.131 | -0.004 | 0.119 | 0.170 | -0.004 | 0.153 |
|  |  |  | **> 50 yrs** | |  |  |
| *E/I ratio* | 0.037 | -0.318 | 0.249 | 0.050 | -0.318 | 0.219 |
| *Valsalva test* | 0.126 | -0.190 | 0.038 | 0.000 | -0.116 | 0.135 |
| *Orthostatism test* | 0.044 | -0.192 | -0.136 | 0.147 | -0.204 | 0.072 |
| *∆SBP* | -0.070 | 0.120 | -0.025 | 0.031 | -0.010 | 0.025 |
| *Total Ewing score* | 0.128 | **-0.392** | -0.025 | 0.044 | **-0.335** | -0.173 |
| *HFa* | 0.091 | 0.177 | 0.090 | 0.067 | 0.022 | -0.160 |
| *LFa* | 0.034 | 0.215 | 0.163 | 0.127 | 0.069 | -0.268 |
| **Women** |  |  | **≤ 50 yrs** | |  |  |
| *E/I ratio* | -0.027 | 0.84 | 0.112 | -0.034 | -0.162 | 0.104 |
| *Valsalva test* | **-0.253** | 0.173 | 0.088 | 0.005 | -0.075 | 0.076 |
| *Orthostatism test* | 0.032 | 0.073 | 0.009 | **-0.216** | 0.029 | 0.079 |
| *∆SBP* | -0.086 | **0.243** | 0.200 | **0.278** | -0.123 | **0.294** |
| *Total Ewing score* | **0.200** | 0.179 | 0.081 | 0.064 | 0.163 | 0.152 |
| *HFa* | 0.005 | 0.102 | 0.103 | 0.134 | -0.115 | 0.162 |
| *LFa* | 0.058 | 0.804 | 0.063 | 0.102 | -0.077 | 0.112 |
|  |  |  | **> 50 yrs** | |  |  |
| *E/I ratio* | 0.072 | 0.004 | -0.024 | -0.016 | 0.073 | -0.017 |
| *Valsalva test* | 0.011 | 0.040 | -0.079 | -0.150 | 0.018 | -0.082 |
| *Orthostatism test* | 0.150 | ***-0.476**** | ***-0.340**** | -0.215 | ***0.444**** | ***-0.329**** |
| *∆SBP* | 0.194 | 0.109 | 0.026 | 0.244 | 0.128 | 0.008 |
| *Total Ewing score* | 0.019 | -0.133 | -0.38 | -0.063 | -0.071 | 0.064 |
| *HFa* | 0.022 | 0.113 | -0.201 | 0.024 | 0.127 | -0.207 |
| *LFa* | -0.006 | 0.163 | -0.272 | 0.051 | 0.209 | -0.291 |

Data are Spearman’s rho coefficient of correlation. Data on bold-face denotes statistically significant correlations (*P* < 0.050). Twenty-nine women taking hormonal contraceptives were not included into these analyses.
